# Inhibitors of the Influenza A Virus M2 Proton Channel Discovered Using a High-Throughput Yeast Growth Restoration Assay

**DOI:** 10.1371/journal.pone.0055271

**Published:** 2013-02-01

**Authors:** Aruna D. Balgi, Jun Wang, Daphne Y. H. Cheng, Chunlong Ma, Tom A. Pfeifer, Yoko Shimizu, Hilary J. Anderson, Lawrence H. Pinto, Robert A. Lamb, William F. DeGrado, Michel Roberge

**Affiliations:** 1 Department of Biochemistry and Molecular Biology, University of British Columbia, Vancouver, British Columbia, Canada; 2 Department of Pharmaceutical Chemistry, University of California San Francisco, San Francisco, California, United States of America; 3 Department of Neurobiology and Physiology, Northwestern University, Evanston, Illinois, United States of America; 4 Department of Biochemistry, Molecular Biology and Cell Biology, Northwestern University, Evanston, Illinois, United States of America; 5 Howard Hughes Medical Institute, Northwestern University, Evanston, Illinois, United States of America; 6 The Centre for Drug Research and Development, Vancouver, British Columbia, Canada; Mount Sinai School of Medicine, United States of America

## Abstract

The M2 proton channel of the influenza A virus is the target of the anti-influenza drugs amantadine and rimantadine. The effectiveness of these drugs has been dramatically limited by the rapid spread of drug resistant mutations, mainly at sites S31N, V27A and L26F in the pore of the channel. Despite progress in designing inhibitors of V27A and L26F M2, there are currently no drugs targeting these mutated channels in clinical trials. Progress in developing new drugs has been hampered by the lack of a robust assay with sufficient throughput for discovery of new active chemotypes among chemical libraries and sufficient sensitivity to provide the SAR data essential for their improvement and development as drugs. In this study we adapted a yeast growth restoration assay, in which expression of the M2 channel inhibits yeast growth and exposure to an M2 channel inhibitor restores growth, into a robust and sensitive high-throughput screen for M2 channel inhibitors. A screen of over 250,000 pure chemicals and semi-purified fractions from natural extracts identified 21 active compounds comprising amantadine, rimantadine, 13 related adamantanes and 6 non-adamantanes. Of the non-adamantanes, hexamethylene amiloride and a triazine derivative represented new M2 inhibitory chemotypes that also showed antiviral activity in a plaque reduction assay. Of particular interest is the fact that the triazine derivative was not sufficiently potent for detection as an inhibitor in the traditional two electrode voltage clamp assay for M2 channel activity, but its discovery in the yeast assay led to testing of analogues of which one was as potent as amantadine.

## Introduction

Influenza A viruses are highly infectious pathogens responsible for seasonal epidemics and for pandemics. Worldwide, seasonal epidemics result in 3–5 million cases of severe illness, and 250,000–500,000 deaths yearly [1], while pandemics such as the 1918 Spanish Flu, 1957 Asian Flu, 1968 Hong Kong Flu, and 2009 Swine Flu have resulted in millions of deaths [2], [3], [4].

Vaccination is the primary strategy for prevention, but antiviral agents are needed to manage seasonal influenza in vulnerable patients and are essential if generation of an appropriate vaccine is not rapid enough during a new pandemic. Only four drugs are currently approved in the USA for influenza A treatment: the viral neuraminidase inhibitors oseltamivir and zanamivir and the viral M2 proton channel inhibitors amantadine and its methyl derivative rimantadine [5]. Of these agents, only amantadine, rimantadine and oseltamivir are orally administered. Strains resistant to the M2 inhibitors are now predominant [6], [7] and resistance to oseltamivir is increasingly encountered [8], [9], [10]. Emergence of strains with resistance to all approved drugs is a distinct possibility and could have particularly serious repercussions in the event of a new pandemic.

Progress is being made in developing new neuraminidase inhibitors [11] but there has been less progress with M2 proton channel inhibitors [12]. The M2 proton channel is required for virus replication and maturation. After the virus is taken up into the host cell by endocytosis, the low pH of the endosome activates the M2 channel to allow proton flux from the endosome into the viral interior. This acidification dissociates the viral RNA from its bound matrix proteins and permits release of the viral genetic material to the cytoplasm for replication [13]. The M2 protein also equilibrates the pH gradient between the Golgi lumen and the cytoplasm to prevent premature conformational changes of hemagglutinin during viral maturation [14], [15]. M2 is a homotetramer with each chain consisting of a short unstructured extracellular N-terminal domain (residues 1–24) that is important for incorporation into the virion; a single transmembrane domain (25–46) that is necessary and sufficient for tetramerization, proton conductance and drug binding; an amphiphilic membrane-associated α-helix (residues 47–61) that is important for viral budding and scission; and a unstructured C-terminal cytoplasmic tail (residues 62–97) that interacts with matrix protein M1 [16]. Amantadine binds the transmembrane region with its charged amino group mimicking hydronium [17]. Because the proton conductance rate of the channel has to match the pH sensitivity of hemagglutinin [14], [18], of the large number of amantadine-resistant mutations that have been identified *in vitro*, only three have been identified in transmissible viruses [19], [20]. These are single amino acid substitutions in the channel - V27A, L26F or S31N.

The development of inhibitors that are active against amantadine-resistant channels is highly desirable but it is very challenging to produce and assay the M2 membrane protein under native-like conditions. Methods of assaying ion channel activity are labor intense and technically demanding [21], [22], [23] and therefore unsuitable for high-throughput screens of chemical libraries. Krystal et al. showed that when the M2 channel gene was expressed in the yeast *Saccharomyces cerevisiae*, a proton-selective channel was formed that disrupted the electrochemical potential across the yeast membrane and inhibited yeast growth, and that compounds that block M2 channel activity were able to restore growth [24]. The same principle was also applied using *E. Coli* but was not implemented for high throughput screening [25]. In this study, we developed the yeast growth restoration assay into a high-throughput screen for inhibitors of the M2 channel. We used it to discover not only additional analogs of known M2 channel inhibitors, but also amiloride derivatives and substituted triazines that represent chemotypes previously unrecognized as M2 channel inhibitors and that provide new starting points for influenza drug development.

## Results

### Development and Validation of a Yeast Growth Restoration Assay to Detect Inhibitors of the A/M2 Channel

**Figure 1:**
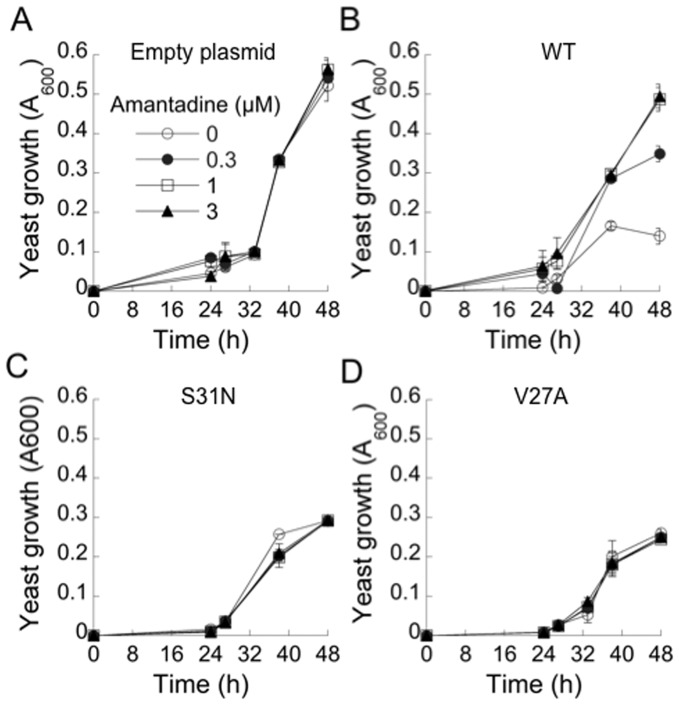
Effect of M2 expression and amantadine on yeast growth. Yeast strains containing an empty plasmid (A) or plasmid bearing WT M2 (B), S31N M2 (C) or V27A M2 (D) were distributed into 96-well plates and their growth was measured over time following transfer at 0h to medium containing galactose and addition of the indicated concentrations of amantadine.

*S. cerevisiae* strains were generated containing a multicopy plasmid for expression of the wild-type, S31N-mutated, or V27A-mutated M2 gene from the Udorn strain of influenza A controlled by the inducible *GAL1* promoter (designated WT, S31N and V27A respectively), or an empty plasmid. The growth of the four strains was monitored over time by turbidimetry following induction of the *GAL1* promoter by galactose. Expression of WT M2 considerably slowed yeast growth to 27% of the growth of the control strain at 48 h (Fig. 1A, B). Expression of amantadine-resistant S31N M2 reduced yeast growth to 60% of the control strain while expression of V27A M2 reduced growth to 55% of control (Fig. 1C, D). Amantadine was used to test whether the observed growth inhibition was caused by M2 proton channel activity. The growth of the yeast strain containing the empty plasmid was not affected by amantadine at 0.3, 1 or 3 µM (Fig. 1A). By contrast, amantadine considerably increased the growth of the strain expressing WT M2 from 27% of control without amantadine to 65% of control at 0.3 µM amantadine, and 95% of control at 1 µM and 3 µM (Fig. 1B). Amantadine did not increase the growth of the S31N and V27A strains (Figs 1C, 1D), as expected since these mutated channels are amantadine-resistant.

**Figure 2:**
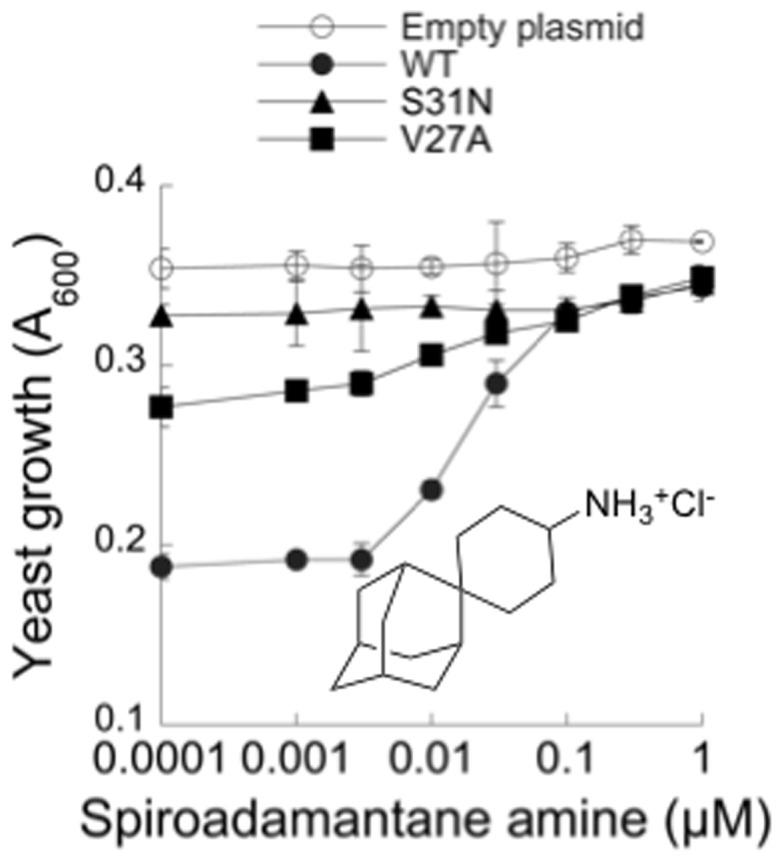
Effect of a spiroadamantane amine on the growth of yeast expressing WT and mutated M2. Yeast strains containing the indicated plasmids were distributed in 96-well plates in medium containing galactose and exposed to the indicated concentrations of the spiroadamantane amine (structure shown) for 40 h.

To test whether growth inhibition by amantadine-resistant M2 mutants was also dependent on proton channel activity, we used a spiroadamantane amine (Fig. 2). This compound inhibits the V27A mutant channel as well as the WT channel in the two electrode voltage clamp (TVEC) assay of M2 channel conductance with IC_50_ of 0.3 and 18.7 µM respectively, and also inhibits replication of recombinant viruses bearing the V27A mutation in a plaque reduction assay [26]. As discussed previously, these assays are conducted at a short time scale of 2 min acidification (due to the limited stability of oocytes at low pH), prior to the establishment of equilibrium. The IC_50_ values obtained with the TEVC assay are therefore one or two orders of magnitude larger than in cellular assays that are conducted at longer time scales [27]. In accordance with these expectations, spiroadamantane amine clearly increased growth of the V27A strain at 0.01 µM and above, with an EC_50_ of 0.3 µM (Fig. 2). It also restored growth of the WT M2 strain in the same concentration range (Fig. 2). Interestingly, the spiroadamantane amine was 3-fold more potent against the WT M2 strain (EC_50_ = 0.03 µM) (Fig. 2) than amantadine (EC_50_ = 0.1 µM).

These results with drug-sensitive and drug-resistant mutants and a mutant-selective inhibitor showed that yeast growth restoration results from inhibition of M2 proton channel activity. Moreover, the ability of low concentrations of the inhibitors to significantly restore growth indicated the potential of this assay for development into a sensitive screen for M2 inhibitors.

### High-throughput Screen for M2 Inhibitors

**Figure 3:**
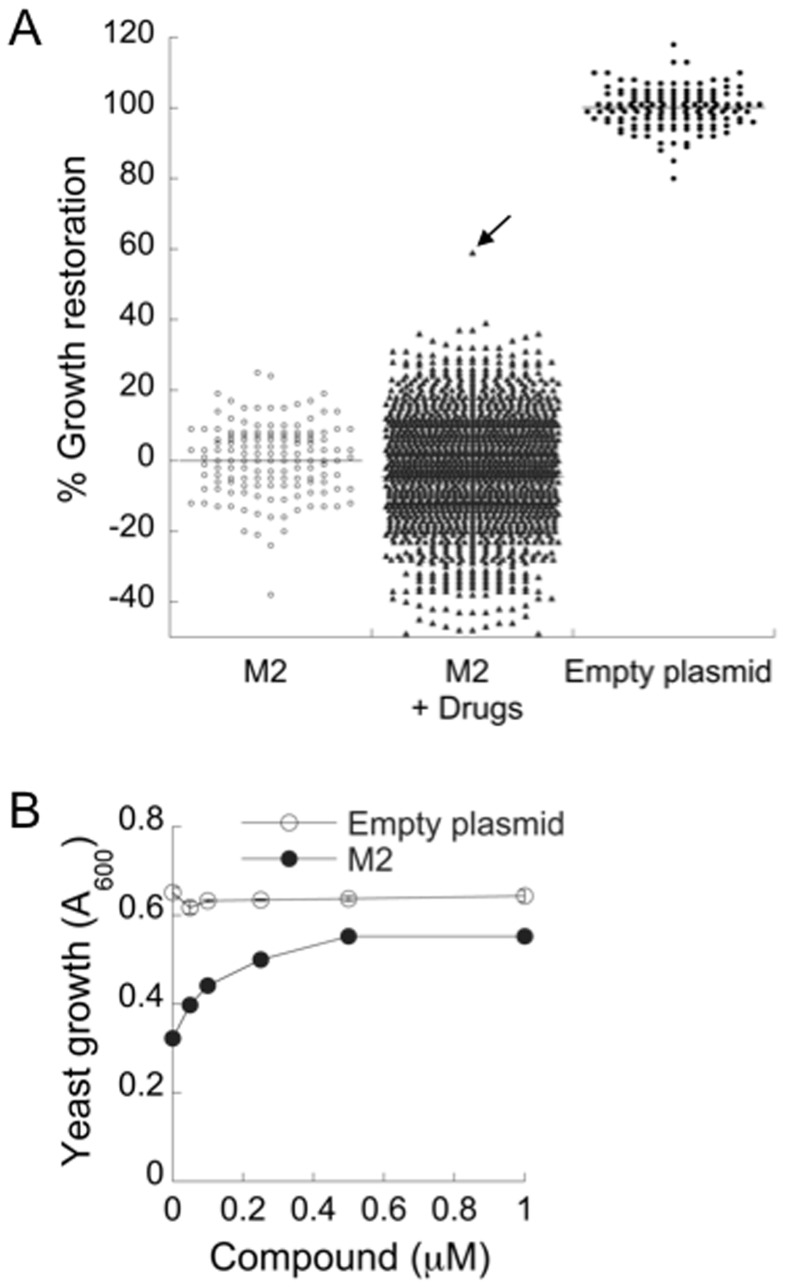
96-well pilot screen results. (A) Yeast expressing WT M2 were exposed to 1,440 screening chemicals (M2+ Drugs) with 8 untreated wells per plate as negative controls (M2, n = 144). Each plate also contained 8 positive controls not treated with drugs (Empty plasmid, n = 144). Growth was measured after 40 h and the % growth restoration was calculated as described in Materials and Methods. The arrow points to the single active compound found in this batch. (B) the active compound found in A was retested at different concentrations against yeast expressing WT M2 or bearing the empty plasmid and growth was measured after 40 h.

To determine whether this assay could be used to discover new inhibitors, a simple benchtop 96-well plate assay was developed (see Materials and Methods). This assay showed good discrimination between the growth of strains expressing WT M2 or empty plasmid (Fig. 3A), with an acceptable Z’ factor of 0.50 [28].

**Figure 4:**
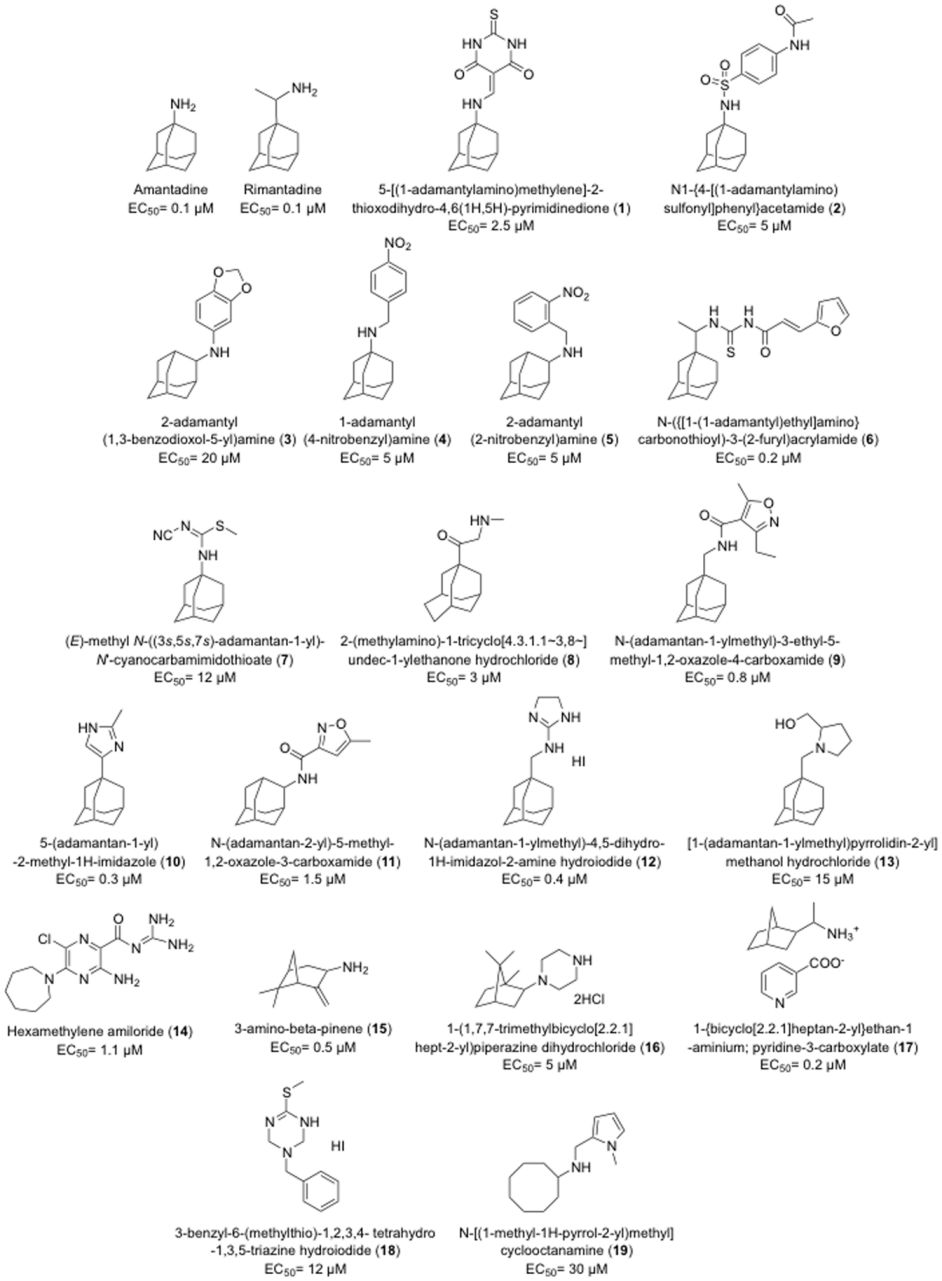
Structures, names and EC_50_ of active compounds found in the screen.

We used this assay in a pilot screen of ∼30,000 commercially available pure chemicals (Prestwick, BioMol, Sigma LOPAC, Microsource Spectrum, Maybridge Hitfinder and ChemBridge DIVERset). A representative example of a screen of a batch of 1,440 compounds and their corresponding control wells tested on the same day is shown in Fig. 3A. Many of the compounds inhibited yeast growth but only one showed substantial growth restoration. This compound, rimantadine derivative **6**, had no effect on the growth of the yeast strain carrying an empty plasmid but it increased the growth of the WT M2 strain in a concentration-dependent manner with an EC_50_ of 0.2 µM (Fig. 3B). Overall, this pilot screen identified 14 active compounds (**1–8**, **14–16**, **18**) as well as amantadine and rimantadine (Fig. 4).

Encouraged by these findings, we sought to increase the capacity and automation of this assay to enable high-throughput screening. A similar assay using 384-well plates and automated liquid handling and plate reading was established. In this assay, yeast expressing WT M2 treated or not with amantadine was used to establish the assay Z’ factor, which averaged 0.54. This assay was used to screen 50,000 pure compounds from Chembridge, yielding 7 additional active compounds (**9–13**, **17**, **19**) (Fig. 4). To enable discovery of structurally novel compounds, we also carried out a screen of 176,000 semi-purified fractions from the Nature Bank collection of natural products extracts [29]. These fractions, which typically contain several compounds, were tested at a dilution expected to result in individual compounds being present at 0.5–5 µM, assuming a MW of 300. Few fractions showed any level of growth restoration. Nevertheless, ∼500 fractions showing >1.8-fold increase in growth (1 µM amantadine caused a 3-fold growth increase) were retested but none showed activity.

### Characterization of Active Compounds

The yeast growth restoration screen identified 21 active compounds, including amantadine, rimantadine, 13 adamantanes and 6 non-adamantanes. The activity of these compounds was confirmed in concentration curves and their names, structures and EC_50_ values are shown in Fig. 4. The EC_50_ of these active compounds ranged from 0.1 to 30 µM. Four adamantanes (**6**, **9**, **10** and **12**) and two non-adamantanes (**15** and **17**) had EC_50_ values below 1 µM.

**Figure 5:**
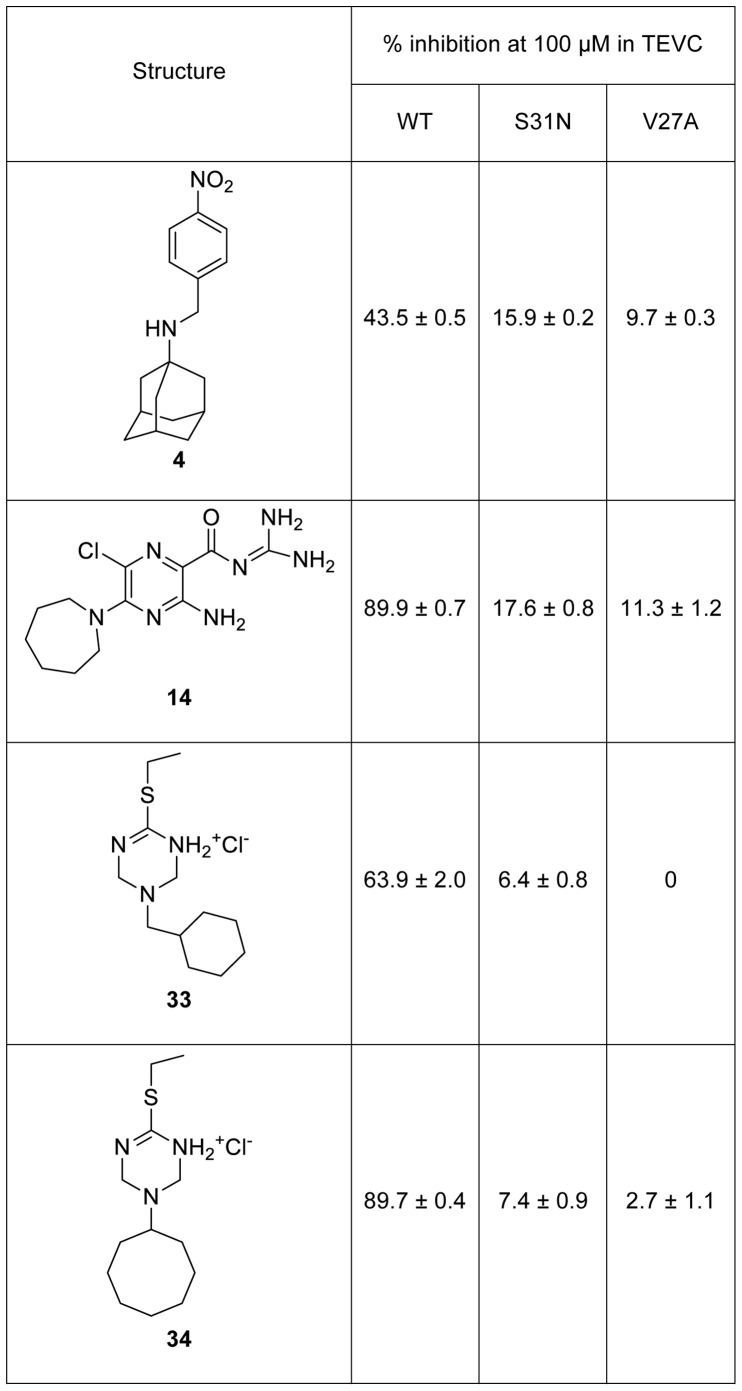
Activity of hexamethylene amiloride and selected triazine analogs in the TEVC assay. Each point is the mean and standard deviation of five to eight oocytes.

All of the compounds with the exception of the weak inhibitor **18** had the bipartite structure of previous M2 inhibitors which includes a hydrophobic scaffold (e.g., adamantane, bicycloheptane, or cyclooctane) and a polar head group [30], [31], [32]. Examination of the active adamantanes (Fig. 4) shows that a wide variety of substitutions are tolerated at either the 1 or 2 position of adamantane, including substituted benzyl (**2**, **3**, **4** and **5**), five-membered heterocycles (**6**, **9**, **10**, **11**, **12** and **13**) and a six-membered heterocycle substitution (**1**). Compounds **3**, **4**, **5**, **8**, **15, 16, 17** and **19** were primary or secondary amines that are likely protonated at the pH of the assay medium (pH = 6.5). This finding is consistent with earlier studies that suggest that the ammonium serves to mimic the hydronium ion in the M2 channel [33]. Additionally, compounds **10**, **12**, **13**, **14** and **18** bear basic heterocycles, acylguanidine or tertiary amines that might also be protonated at near neutral or slightly acidic pH. The remaining compounds **1**, **2**, **6**, **7**, **9** and **11** contain neutral polar groups, particularly cyclic or acyclic thiourea (**1** and **6**) and sulfonamide (**2**), some of them seen in previously reported M2 inhibitors [30], [34]. Compound **4** was selected to be tested in the TEVC assay and found to inhibit 43.5% of the WT channel activity at 100 µM (Fig. 5), which correlates with the yeast screening result with EC_50_ of 5 µM (Fig. 4).

Among the active compounds not containing an adamantane moiety (Fig. 4), bicyclic compounds such as derivatives of pinene **15**, camphor **16** and bicycloheptane **17** had been shown previously to be M2 inhibitors [31], [32], [35] supporting the statement that the M2 WT channel is able to accommodate hydrophobic molecules with cage shapes other than that of adamantane [12]. Pyrrole-substituted cyclooctylamine **19** was active, but with a poor EC_50_ of 30 µM. Cyclooctylamine had been found by Shroeder’s group to block M2 channel proton flux in a liposome assay [36] and its binding affinity was later determined to be 6-fold weaker than amantadine [37]. Together, these results show that WT M2 channel is able to accommodate compounds with diverse structures.

Significantly, two active compounds, hexamethylene amiloride **14** and triazine **18** (EC_50_ of 1.1 and 12 µM respectively), have not been reported previously as M2 inhibitors. To determine whether restoration of yeast growth by these compounds was truly a result of M2 channel inhibition, we purchased or synthesized several analogs of **14** and **18** and tested them in the TEVC assay, which directly measures proton channel conductance.

Hexamethylene amiloride **14** was highly active against WT M2 with 89.9% channel conductance inhibition at 100 µM, as active as amantadine (Fig. 5). Three close structural analogs **20**, **21** and **22** were also tested and these showed only moderate inhibition (Table S1). However, a clear structure-activity relationship was observed in this series of amiloride analogs - the more hydrophobic the molecule is, the more potent it is in inhibiting M2. This was anticipated based on earlier SAR results that all active compounds have cLogP>1.5 [30].

Triazine **18** was not active (4.5%) in the TEVC assay at 100 µM (Table S1). Twelve closely related structural analogs of **18** (compounds **23**–**34)** were tested in the TVEC assay. Interestingly, the two compounds with hydrophobic scaffolds of more than seven carbon atoms were active (Fig. 5) while all others were essentially inactive in this assay (<20% inhibition at 100 µM) (Table S1). Active compounds **33** and **34** were tested in the yeast growth restoration assay and showed EC_50_ values of 0.7 µM and 0.3 µM respectively, compared with an EC_50_ of 12 µM for triazine **18.** Given the good correspondence between inhibition at 100 µM in the TEVC assay and potency in the yeast assay for compounds identified here, and the fact that the TEVC assay requires much higher inhibitor concentrations than the yeast assay to show activity, we surmise that triazine **18** is not a false positive from the yeast assay but rather a true M2 inhibitor that is not sufficiently potent to be detected by the TVEC assay. Taken together, these results point to the importance of the contribution of the hydrophobic scaffolds to the high activity of the inhibitors (compound **14**, **33** and **34**) of WT M2 rather than the polar headgroup, which explains why a wide variety of polar headgroups can be tolerated.

Given the high prevalence of amantadine-resistant mutations in seasonal flu strains, it was of interest to determine whether any of the compounds found to be active against WT M2 also showed activity towards the S31N or V27A mutant channels. Compounds **1–19**, **33** and **34** were tested against these two mutants in the yeast assay. Compound **16** showed significant activity against the V27A mutant (EC_50_ = 7 µM) and was inactive against S31N while all other compounds were inactive against V27A or S31N. Compounds **14**, **18**, and **20–34** were also tested at 100 µM against the S31N and V27A mutant channels in the TEVC assay and were found to be inactive (Fig. 5 and Table S1).

**Figure 6:**
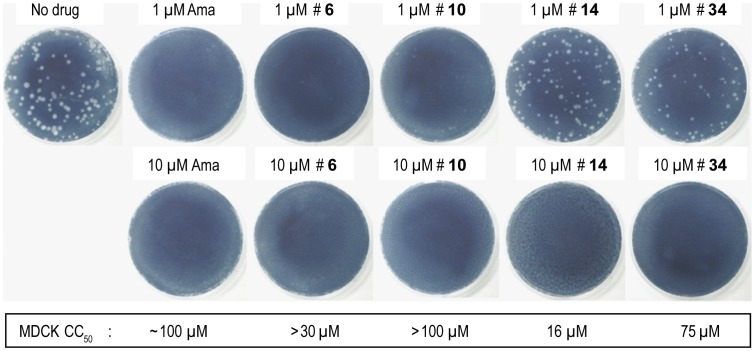
Antiviral activity and cytotoxicity of selected compounds. The effect of the compounds on influenza A/Udorn/72 WT virus replication was evaluated by plaque formation in MDCK cells. Cytotoxicity towards MDCK cells was assayed after 48 h drug exposure with the MTT tetrazolium dye assay as described [55].

Two potent adamantanes (**6** and **10**) and two non-adamantanes (**14**, **34)** were further tested for inhibition of influenza A replication in a MDCK plaque reduction assay [27], with amantadine as a positive control. All four compounds completely inhibited plaque formation at 10 µM (Fig. 6). At 1 µM, compounds **6** and **10** almost completely inhibited plaque formation, comparable to 1 µM amantadine, compound **34** showed moderate inhibition, and compound **14** had nearly no effect (Fig. 6). The potency of the antiviral effect of **6**, **10** and **34** was consistent with their low EC_50_ in the yeast growth restoration assays (0.2 µM, 0.3 µM and 0.3 µM, respectively), compared with an EC_50_ of 0.1 µM for amantadine. The good potency of compound **34** agreed with its TEVC assay result showing 89.7% inhibition at 100 µM (92.0% for amantadine at 100 µM). Hexamethyleneamiloride **14,** which was less potent in the yeast assay (EC_50_ = 1.1 µM) was also less potent in the plaque reduction assay. The four compounds were also tested for cytotoxicity towards uninfected MDCK cells. Compounds **6**, **10** and **34** showed very low cytotoxicity, indicative of a good activity window, while hexamethyleneamiloride was more cytotoxic, with a CC_50_ of 16 µM (Fig. 6), indicative of a less favorable activity window. Overall, the results demonstrate that the yeast growth restoration assay can identify M2 inhibitors with antiviral activity.

## Discussion

Progress in finding new inhibitors targeting drug resistant M2 channels has been slow but recent advances in understanding the structure and dynamic properties of the M2 channel in a lipid bilayer environment [38], [39], [40], and the interaction of amantadine with the channel [41], [42], [43], [44] have spurred structure-based drug design and virtual screening efforts. Several new inhibitors were described in the last few years, many closely resembling amantadine [12]. However, inhibitors targeting drug resistant M2 mutants have not been reported. A notable exception is the spiroadamantane amine shown in Fig. 2 that is active against WT, L26F and V27A M2 [26]. A robust and sensitive high-throughput screening assay would enable sampling of an expanded chemical diversity repertoire and guide SAR studies.

In this study, we modified an assay described by Krystal et al [24] and optimized it for high-throughput screening, notably by expressing M2 in a yeast strain with defective drug efflux systems [45], and by simplifying and automating assay conditions. The resulting growth restoration assay is robust, highly sensitive, quantitative, cost-effective and technically simple. We found that the yeast growth inhibition was greatest for WT, giving rise to a good Z’ factor for screening. Because a successful drug should inhibit WT M2 as well as amantadine-resistant mutated forms, we use WT in the primary screen followed by testing of active compounds against mutants in secondary assays. Our screen of over 250,000 pure chemicals and semi-purified fractions from natural extracts identified only 21 active compounds. This 0.008% hit rate is 10- to 100-fold lower than typically encountered in screens for other targets. The assay shows high selectivity for M2 channel inhibitors as further testing of the 21 active compounds revealed no false positives. In positive readout cell-based assays, high selectivity is imparted largely by the requirement for active compounds to both inhibit their target and not hamper cell growth, effectively eliminating many non-selective active chemicals. However, since we have observed higher hit rates in yeast growth restoration assays with other targets [46], the very low hit rate and the paucity of chemical classes identified indicates that M2 is not easily inhibited by the drug-like synthetic chemicals assembled in commercially available libraries. Natural products are known to encompass a much wider range of biologically relevant chemical space, so we were surprised that our large-scale screen of semi-purified fractions obtained from natural extracts did not yield a single hit. We do not believe this is due to technical factors such as testing of the fractions at too low a concentration to reveal activity [47]. Rather, we think this indicates that the M2 channel can be selectively inhibited by very few pharmacophores and that it is a challenging target for drug development.

Nonetheless, the screen identified two compounds with novel structures for M2 channel inhibitors – hexamethylene amiloride **14** and triazine **18**. Further testing in the TEVC assay confirmed that hexamethylene amiloride **14** inhibited the M2 channel, and subsequent SAR evaluation of amiloride analogs showed that hydrophobicity played a critical role in M2 channel inhibition. Hexamethylene amiloride **14** is a known inhibitor of the mammalian Na^+^/H^+^ exchanger [48], and is also active against the HCV proton channel p7, the HIV-1 Vpu ion channel and the SARS Coronavirus envelope protein ion channel activity [49], [50], [51]. Hexamethylene amiloride **14** inhibits the mammalian Na^+^/H^+^ exchanger with an apparent K_i_ of 0.2 µM [52], more potently than it inhibits M2 in the yeast assay (EC_50_ = 1 µM) and therefore does not appear to be a good drug candidate. An azolo-1,2,4-triazine derivative has been described as an inhibitor of influenza A and B virus replication [53], with an uncharacterized mechanism of action. Triazines **18**, **33** and **34** described here are considerably different and have not previously been linked to the inhibition of ion channels. Triazine 34 completely inhibited influenza A replication at 10 µM and was not very toxic to MDCK cells (CC_50_ = 75 µM). These compounds may be good starting points for drug discovery. The high sensitivity of this yeast-based assay also revealed that the spiroadamantane amine shown in Fig. 2 is not only highly potent against the amantadine-resistant V27A mutant as reported [26], but that it is a 3-fold more potent inhibitor of the WT M2 channel than amantadine and rimantadine, and may therefore be a candidate for drug development. Together, these results demonstrate the value of the yeast growth restoration assay for high throughput screening of M2 inhibitors and discovery of anti-influenza agents.

## Materials and Methods

### Yeast Strains and Growth Measurement

The multicopy expression plasmids, the yeast strains and the assay to measure yeast growth used in this study are described in detail in a previous publication [45]. The coding sequences of the Udorn WT, S31N and V27A M2 genes were introduced downstream of the GAL1 promoter by PCR amplification followed by recombination cloning in yeast. Plasmids were rescued from the yeast strains and coding sequences were sequence-verified. The yeast strains were propagated in synthetic complete (SC) medium pH 6.5 lacking leucine and containing glucose to repress M2 gene expression. To measure growth under M2 expression conditions, yeast were harvested by centrifugation, the pellets were washed with water to remove traces of glucose and they were suspended to A_600_ = 0.008 in liquid SC medium pH 6.5 lacking leucine and containing 2% galactose. 100 µl samples were distributed into 96-well plates and incubated at 30°C in a humidified chamber, without agitation. A_600_ was measured in a microplate reader at different times after suspending the cells by gentle but thorough vortexing.

### 96-well Medium-throughput Screening Assay

WT M2 yeast were grown as above, suspended at A_600_ = 0.01 in liquid SC medium without leucine and with 2% galactose, and distributed at 100 µl per well into sterile transparent 96-well plates using 8-channel dispensing pipetors. Screening chemicals were transferred from 5 mM stock solutions in DMSO to yeast plates at a final concentration of ∼15 µM using a Biorobotics TAS1 robot equipped with a 0.7 mm diameter 96-pin tool. The plates were incubated at 30°C in a humidified incubator. After 40–42 h cells were suspended by vortexing and A_600_ was measured. The percentage of growth restoration was calculated as described [45]. Chemicals showing growth restoration were retested in triplicate at different concentrations against WT M2 and empty plasmid strains to confirm activity and determine EC_50_.

### 384-well Plate High throughput Screening Assay

The 96-well plate assay was adapted to 384-well format as follows: 30 µl of SC medium lacking leucine and containing 2% galactose was distributed into wells of Corning #3680 clear polystyrene 384-well plates using a Matrix Wellmate microplate dispenser. Screening chemicals and extracts were added using a Matrix Platemate Plus automated liquid handling system equipped with a 0.7 mm diameter FP3 384-pin tool transferring ∼80 nl. 20 µl yeast at A_600_ = 0.00625 in the same medium was then added using the matrix Wellmate dispenser. The plates were incubated at 30°C in a humidified Fisher isotemp incubator for 44 h and A_600_ was measured without shaking using a Biotek Powerwave plate reader integrated with a Biotek Biostack plate stacking system. Plates were assayed in batches of 40–50 plates. Each 384-well plate contained 16 control wells not exposed to drug and 16 control wells into which 1.8 µM amantadine had been dispensed using the Matrix Wellmate.

### TVEC Assay

Compounds were tested via a two-electrode patch clamp assay using *Xenopus laevis* oocytes microinjected with RNA for expression of the WT, S31N or V27A M2 gene [27].

### Chemical Synthesis

Compounds listed in Fig. 5 and Table S1 were obtained either from commercial sources and used without further purification or synthesized according to a literature procedure [54]. The following compounds were ordered from Sigma-Aldrich: **14** (cat. # A9561), **20** (cat. # A4562), **21** (cat. # A6085), **22** (cat. # A5585). The following compounds were ordered from Maybridge: **18** (cat. # DP01558), **25** (cat. # DP01601), **26** (cat. # DP01622), **27** (cat. #BTB03080), **29** (cat. #BTB03554). Compounds **23**, **24**, **28**, **30**, **31**, **32**, **33** and **34** were synthesized according to the procedure illustrated in Fig. S1. Amine (1 mmol) was mixed with formaldehyde (2 mmol) in dioxane (5 ml). Isothiourea (1 mmol) was subsequently added. The mixture was heated until a clear solution was formed and allowed to stir at room temperature overnight. The mixture was extracted with dichloromethane and saturated NaHCO_3_ solution. The organic phase was combined and dried over MgSO_4_, filtered and the solvents were removed under reduced pressure. The crude product was purified by silica gel flash column chromatography with a gradient of 10–20% CH_3_OH/dichloromethane. The synthesized compounds were characterized by ^1^HNMR and MS. Selected compounds were also characterized with ^13^CNMR.

6-(methylthio)-3-(thiophen-2-ylmethyl)-1,2,3,4-tetrahydro-1,3,5-triazine (**23**). ^1^HNMR (500 MHz, CD_3_OD): δ 7.42–7.41 (m, 1H), 7.11–7.10 (m, 1H), 7.00–6.98 (m, 1H), 4.51 (s, 4H), 4.16 (s, 2H), 2.66 (s, 3H). ^13^CNMR (125 MHz, CD_3_OD): 166.54, 140.55, 129.04, 127.97, 127.67, 62.12, 50.96, 13.37. EI-MS: *m*/*z* (M+H^+^): 228.4 (calculated), 228.4 (found).

6-(ethylthio)-3-(furan-2-ylmethyl)-1,2,3,4-tetrahydro-1,3,5-triazine (**24**). ^1^HNMR (500 MHz, CD_3_OD): δ 7.51–7.50 (m, 1H), 6.42–6.41 (m, 2H), 4.48 (s, 4H), 3.95 (s, 2H), 3.17 (q, *J* = 7.0 Hz, 2H), 1.40 (t, *J* = 7.0 Hz, 3H). ^13^CNMR (125 MHz, CD_3_OD): 165.03, 151.93, 144.52, 111.72, 111.03, 62.49, 50.96, 26.16, 14.66. EI-MS: *m*/*z* (M+H^+^): 226.3 (calculated), 226.3 (found).

3-benzyl-6-(ethylthio)-1,2,3,4-tetrahydro-1,3,5-triazine (**28**). ^1^HNMR (500 MHz, CD_3_OD): δ 7.42–7.32 (m, 5H), 4.46 (s, 4H), 3.90 (s, 2H), 3.21 (q, *J* = 7.0 Hz, 2H), 1.42 (t, *J* = 7.0 Hz, 3H). ^13^CNMR (125 MHz, CD_3_OD): 164.98, 137.95, 130.37, 129.90, 129.27, 62.63, 56.39, 26.24, 14.76. EI-MS: *m*/*z* (M+H^+^): 236.4 (calculated), 236.3 (found).

6-(ethylthio)-3-(thiophen-2-ylmethyl)-1,2,3,4-tetrahydro-1,3,5-triazine (**29**). ^1^HNMR (500 MHz, CD_3_OD): δ 7.42–7.40 (m, 1H), 7.10–7.08 (m, 1H), 7.00–7.98 (m, 1H), 4.93 (s, 4H), 4.12 (s, 2H), 3.21 (q, *J* = 7.0 Hz, 2H), 1.42 (t, *J* = 7.0 Hz, 3H). ^13^CNMR (125 MHz, CD_3_OD): 165.11, 140.95, 128.80, 127.93, 127.58, 62.22, 51.00, 26.26, 14.73. EI-MS: *m*/*z* (M+H^+^): 242.4 (calculated), 242.2 (found).

6-(ethylthio)-3-(2-(thiophen-2-yl)ethyl)-1,2,3,4-tetrahydro-1,3,5-triazine (**30**). ^1^HNMR (500 MHz, CD_3_OD): δ 7.21–7.20 (m, 1H), 6.94–6.90 (m, 2H), 4.47 (s, 4H), 3.19 (q, *J* = 7.0 Hz, 2H), 3.11 (t, *J* = 7.0 Hz, 2H), 3.00 (t, *J* = 7.0 Hz, 2H), 1.39 (t, *J* = 7.0 Hz, 3H). ^13^CNMR (125 MHz, CD_3_OD): 165.02, 142.84, 127.91, 126.54, 124.96, 68.25, 63.51, 54.17, 29.85, 26.24, 14.73. EI-MS: *m*/*z* (M+H^+^): 256.4 (calculated), 256.4 (found).

6-(ethylthio)-3-isobutyl-1,2,3,4-tetrahydro-1,3,5-triazine (**31**). ^1^HNMR (500 MHz, CD_3_OD): δ 4.45 (s, 4H), 3.20 (q, *J* = 7.5 Hz, 2H), 2.48 (d, *J* = 7.0 Hz, 2H), 1.85–1.80 (m, 1H), 1.40 (t, *J* = 7.0 Hz, 3H), 0.96 (d, *J* = 6.5 Hz, 6H). ^13^CNMR (125 MHz, CD_3_OD): 164.89, 63.91, 60.44, 28.07, 26.24, 20.84, 14.75. EI-MS: *m*/*z* (M+H^+^): 202.3 (calculated), 202.3 (found).

6-(ethylthio)-3-neopentyl-1,2,3,4-tetrahydro-1,3,5-triazine (**32**). ^1^HNMR (500 MHz, CD_3_OD): δ 4.40 (s, 4H), 3.20 (q, *J* = 7.5 Hz, 2H), 2.49 (s, 2H), 1.41 (t, *J* = 7.5 Hz, 3H), 0.95 (s, 9H). ^13^CNMR (125 MHz, CD_3_OD): 165.32, 66.34, 65.67, 34.07, 27.66, 26.22, 14.73. EI-MS: *m*/*z* (M+H^+^): 216.4 (calculated), 216.3 (found).

3-(cyclohexylmethyl)-6-(ethylthio)-1,2,3,4-tetrahydro-1,3,5-triazine (**33**). ^1^HNMR (500 MHz, CD_3_OD): δ 4.43 (s, 4H), 3.20 (q, *J* = 7.5 Hz, 2H), 2.52 (d, *J* = 7.0 Hz, 2H), 1.92–1.72 (m, 8H), 1.37 (t, *J* = 7.5 Hz, 3H), 1.36–1.11 (m, 3H). EI-MS: *m*/*z* (M+H^+^): 242.4 (calculated), 242.2 (found).

3-cyclooctyl-6-(ethylthio)-1,2,3,4-tetrahydro-1,3,5-triazine (**34**). ^1^HNMR (500 MHz, CD_3_OD): δ 4.83 (s, 4H), 3.17–3.16(m, 1H), 3.14 (q, *J* = 7.5 Hz, 2H), 2.03–1.99 (m, 1H), 1.86–1.82 (m, 4H), 1.67–1.58 (m, 9H), 1.37 (t, *J* = 7.5 Hz, 3H). EI-MS: *m*/*z* (M+H^+^): 256.4 (calculated), 256.3 (found).

## Supporting Information

Figure S1 **Procedure used to synthesize compounds 23, 24, 28, 30, 31, 32, 33 and 34.**
(PDF)

Table S1 **Activity of hexamethylene amiloride and triazine analogs in the TEVC assay.**
(PDF)
