# Supplementary figures and images for: Inhibitors of the Influenza A Virus M2 Proton Channel Discovered Using a High-Throughput Yeast Growth Restoration Assay
Source: PLoS One. 2013 Feb 1;8(2):e55271. doi: 10.1371/journal.pone.0055271 (PMC3562233; doi:10.1371/journal.pone.0055271)

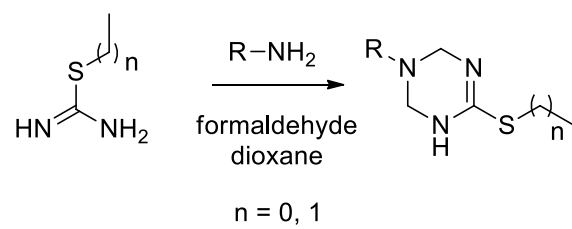

Figure S1: Procedure used to synthesize compounds **23**, **24**, **28**, **30**, **31**, **32**, **33** and **34**.

Supplement: Figure S1 — Procedure used to synthesize compounds 23, 24, 28, 30, 31, 32, 33 and 34. (PDF) [file pone.0055271.s001.pdf]
