# Supplementary material for: Inhibitors of the Influenza A Virus M2 Proton Channel Discovered Using a High-Throughput Yeast Growth Restoration Assay
Source: PLoS One. 2013 Feb 1;8(2):e55271. doi: 10.1371/journal.pone.0055271 (PMC3562233; doi:10.1371/journal.pone.0055271)
Supplement: Table S1 — Activity of hexamethylene amiloride and triazine analogs in the TEVC assay. (PDF) [file pone.0055271.s002.pdf]

Table S1. Activity of hexamethylene amiloride and triazine analogs in the TEVC assay

Compound name (**ID**)	Structure	% inhibition at 100 µM in TEVC		
		WT	S31N	V27A
N-(4-nitrobenzyl)adamantan-1-amine (**4**)	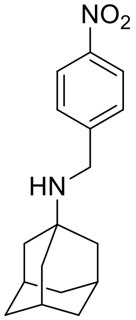	43.5 ± 0.5	15.9 ± 0.2	9.7 ± 0.3
Hexamethylene amiloride (**14**)	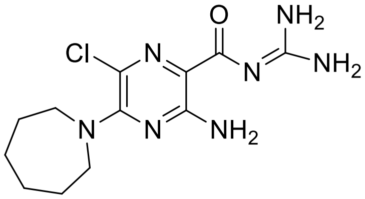	89.9 ± 0.7	17.6 ± 0.8	11.3 ± 1.2
5-(*N*,*N*-Dimethyl)amiloride hydrochloride (**20**)	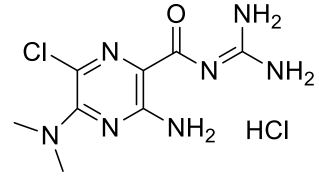	23.3 ± 1.9	0	0
5-(N-Ethyl-N-isopropyl)amiloride (**21**)	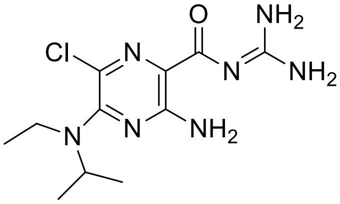	47.3 ± 0.5	5.8 ± 0.6	0
5-(N-Methyl-N-isobutyl)-amiloride (**22**)	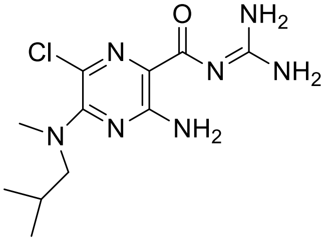	55.7 ± 1.0	1.1 ± 1.1	2.1 ± 2.1
3-benzyl-6-(methylthio)-1,2,3,4- tetrahydro -1,3,5-triazine hydroiodide (**18**)	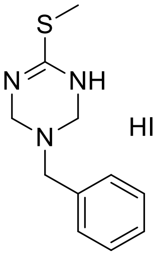	4.5 ± 2.3	3.4 ± 1.7	0

6-(methylthio)-3-(thiophen-2-ylmethyl)-1,2,3,4-tetrahydro-1,3,5-triazin-1-ium chloride (**23**)	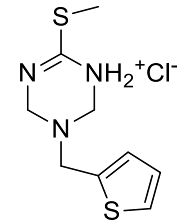	8.1 ± 1.7	1.6 ± 1.6	2.9 ± 1.3
6-(ethylthio)-3-(furan-2-ylmethyl)-1,2,3,4-tetrahydro-1,3,5-triazin-1-ium chloride (**24**)	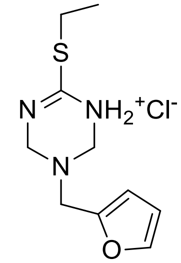	17.9 ± 2.8	1.9 ± 1.6	3.1 ± 2.6
3-(tert-butyl)-6-(methylthio)-1,2,3,4-tetrahydro-1,3,5-triazine hydrobromide (**25**)	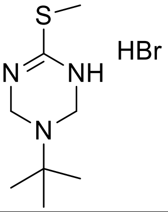	13.3 ± 1.5	7.4 ± 1.2	0
3-(4-(methylthio)-5,6-dihydro-1,3,5-triazin-1(2H)-yl)propan-1-ol hydrobromide (**26**)	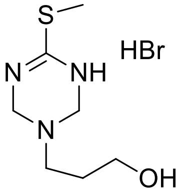	5.5 ± 0.9	4.2 ± 1.0	11.9 ± 1.0
3-(2-methylbut-3-yn-2-yl)-6-(methylthio)-1,2,3,4-tetrahydro-1,3,5-triazine hydroiodide (**27**)	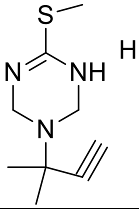	17.0 ± 1.9	4.1 ± 0.2	1.8 ± 1.8
3-benzyl-6-(ethylthio)-1,2,3,4-tetrahydro-1,3,5-triazin-1-ium chloride (**28**)	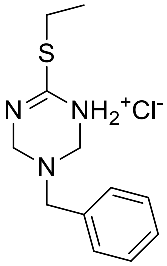	5.4 ± 1.6	4.4 ± 0.7	1.1 0.8
6-(ethylthio)-3-(thiophen-2-ylmethyl)-1,2,3,4-tetrahydro-1,3,5-triazin-1-ium chloride (**29**)	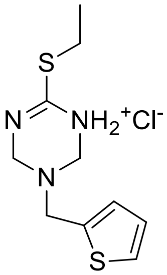	6.7 ± 1.4	6.1 ± 1.2	5.1 ± 0

6-(ethylthio)-3-(2-(thiophen-2-yl)ethyl)-1,2,3,4-tetrahydro-1,3,5-triazin-1-ium chloride (**30**)	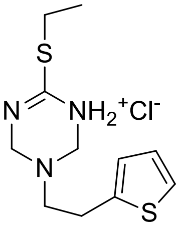	1.1 ± 1.1	8.1 ± 1.0	5.5 ± 1.4
6-(ethylthio)-3-isobutyl-1,2,3,4-tetrahydro-1,3,5-triazin-1-ium chloride (**31**)	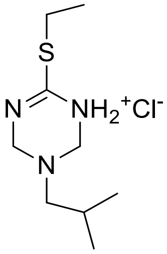	1.2 ± 0.6	3.1 ± 0.5	0.3 ± 0.3
6-(ethylthio)-3-neopentyl-1,2,3,4-tetrahydro-1,3,5-triazin-1-ium chloride (**32**)	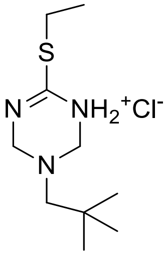	4.7 ± 1.3	2.9 ± 0.4	0.6 ± 0.6
3-(cyclohexylmethyl)-6-(ethylthio)-1,2,3,4-tetrahydro-1,3,5-triazin-1-ium chloride (**33**)	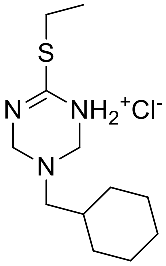	63.9 ± 2.0	6.4 ± 0.8	0
3-cyclooctyl-6-(ethylthio)-1,2,3,4-tetrahydro-1,3,5-triazin-1-ium chloride (**34**)	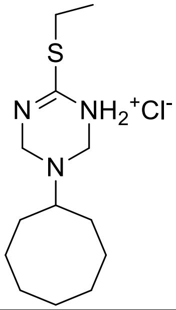	89.7 ± 0.4	7.4 ± 0.9	2.7 ± 1.1
